# Insights into the Structure of the Highly Glycosylated Ffase from *Rhodotorula dairenensis* Enhance Its Biotechnological Potential

**DOI:** 10.3390/ijms232314981

**Published:** 2022-11-29

**Authors:** Elena Jiménez-Ortega, Egle Narmontaite, Beatriz González-Pérez, Francisco J. Plou, María Fernández-Lobato, Julia Sanz-Aparicio

**Affiliations:** 1 Department of Crystallography and Structural Biology, Institute of Physical-Chemistry Rocasolano, CSIC, 28006 Madrid, Spain; 2 Department of Molecular Biology, Centre of Molecular Biology Severo Ochoa, CSIC-UAM, 28049 Madrid, Spain; 3 Institute of Catalysis and Petrochemistry, CSIC, 28049 Madrid, Spain

**Keywords:** β-fructofuranosidase, glycosyl hydrolase family 32, X-ray crystallography, mutagenesis, O-glycosylation, transfructosylation activity, fructooligosaccharides

## Abstract

*Rhodotorula dairenensis* β-fructofuranosidase is a highly glycosylated enzyme with broad substrate specificity that catalyzes the synthesis of 6-kestose and a mixture of the three series of fructooligosaccharides (FOS), fructosylating a variety of carbohydrates and other molecules as alditols. We report here its three-dimensional structure, showing the expected bimodular arrangement and also a unique long elongation at its N-terminus containing extensive O-glycosylation sites that form a peculiar arrangement with a protruding loop within the dimer. This region is not required for activity but could provide a molecular tool to target the dimeric protein to its receptor cellular compartment in the yeast. A truncated inactivated form was used to obtain complexes with fructose, sucrose and raffinose, and a Bis-Tris molecule was trapped, mimicking a putative acceptor substrate. The crystal structure of the complexes reveals the major traits of the active site, with Asn387 controlling the substrate binding mode. Relevant residues were selected for mutagenesis, the variants being biochemically characterized through their hydrolytic and transfructosylating activity. All changes decrease the hydrolytic efficiency against sucrose, proving their key role in the activity. Moreover, some of the generated variants exhibit redesigned transfructosylating specificity, which may be used for biotechnological purposes to produce novel fructosyl-derivatives.

## 1. Introduction

Invertases or β-fructofuranosidases (EC 3.2.1.26) are biotechnologically important enzymes that catalyze the release of β-fructose from the non-reducing termini of various β-D-fructofuranoside substrates. These enzymes may also produce short-chain fructooligosaccharides (FOS) by transfructosylation, in which one to three fructosyl moieties are linked to a sucrose unit [1,2]. FOS are well recognized for their beneficial effects on the organism, such as stimulating the growth of beneficial bacteria in the gut while inhibiting pathogenic bacteria, also known as a prebiotic effect [3]. It also improves the intestinal absorption of minerals and trace elements [4], decreases serum levels of cholesterol, phospholipids and triglycerides, has low carcinogenicity [5,6], reduces the risk of colon cancer [7] and even improves mental health [8,9].

Based on the linkage pattern between monosaccharides, FOS can be characterized into three main groups: levan type, where fructose units are linked by β-(2→6) linkages (^6^F-FOS, e.g., 6-kestose), inulin β-(2→1) bonded type (^1^F-FOS, e.g., 1-kestose), and neoseries fructans where a β-(2→6) linkage connects fructose to the glucosyl moiety of sucrose (^6^G-FOS, e.g., neokestose) [10]. Differences in the chemical structure affect the biological function of FOS, and it appears that β-(2→6) linkages containing FOS (6-kestose) have enhanced potential prebiotic properties as well as chemical stability compared to those traditionally commercialized ^1^F-FOS [3,11,12]. Neokestose has superior prebiotic effects compared to commercial FOS; in addition, it can also prevent colorectal cancer and has an inhibitory effect against melanoma cells [13,14].

β–Fructofuranosidases belong to the glycosyl hydrolase family GH32 (CAZy; http://www.cazy.org, accessed on 25 October 2022), which includes other β–fructofuranosidases, invertases, inulinases and fructosyltransferases. Based on their structural similarities, they are grouped in the GH-J clan, along with the GH68 enzymes [15,16]. Both families share a common five-fold β-propeller domain, whereas GH32 enzymes have an additional C-terminal β-sandwich domain attached to their N-terminal catalytic domain [17]. Three key amino acid residues located in the active site, responsible for substrate binding and hydrolysis, are surrounded by conservative sequences among members of the GH32 family: WMNDPNG (D acting as a nucleophile), FRDP (D acting as a stabilizer of the transient state) and ECP (E acting as an acid-base catalyst) [18,19].

In the last years, the crystallographic structure of various GH32 proteins has been determined, including bacteria [20,21,22], fungi [23,24,25], plants [26,27,28] and animals [29] invertases, all of which appear to be monomeric. However, studies on *Schwanniomyces occidentalis* β-fructofuranosidase (SoFfase) [30], *Saccharomyces cerevisiae* invertase (ScINV) [31], *Xanthophyllomyces dendrorhous* β-fructofuranosidase (XdINV) [2] revealed that yeast enzymes are unique in that they form dimers, mediated by their β-sandwich domain, which in case of ScINV are assembled into an octamer. Although XdINV exhibits a different dimerization pattern compared to the other yeast enzymes, it is clear that the β-sandwich domain is involved in the assembly of the subunits within the different dimers and also in the formation of the active site cavities; moreover, a direct role of the supplementary GH32 domain in substrate binding has been proved [2,31].

The large β–fructofuranosidase from *Rhodotorula dairenensis* RdINV (170 kDa) produces mainly 6-kestose but also a varied mixture of the three series of FOS [1,32], which makes it of biotechnological interest. Additionally, RdINV is also able to fructosylate a variety of carbohydrates, such as disaccharides, alditols and monosaccharides [1]. However, although RdINV has already been biochemically characterized [32] and heterologously expressed in *Komagataella phaffii* [1], the structural determinants responsible for its interaction with substrates and selective production of FOS are unclear. Furthermore, both native and recombinant RdINV showed a molecular mass of ~170 kDa, which is far from the theoretical ~70 kDa calculated on the basis of the deduced sequence of RdINV, and enzymatic treatment with different deglycosylating enzymes revealed that glycans are responsible for 50% of the total molecular mass, with only 15% attributed to N-glycosylation. Interestingly, the predicted O-glycosylation sites are concentrated at the N-terminus of its sequence. Indeed, this is an interesting issue considering that glycosylation plays an important role in protein folding and localization, trafficking, cell–cell interaction or developmental processes. In particular, O-glycosylation of secretory proteins has been found to be an important factor in fungal biology and virulence [33].

In this work, we report the three-dimensional structure of RdINV that discloses the putative biological role of its highly O-glycosylated region and the key amino acid residues responsible for the activity. We present complexes with relevant substrates that allowed us to unveil the substrate binding subsites in the catalytic pocket. The role of identified leading residues has been investigated by site-directed mutagenesis.

## 2. Results and Discussion

### 2.1. RdINV Overall Folding

**Figure 1:**
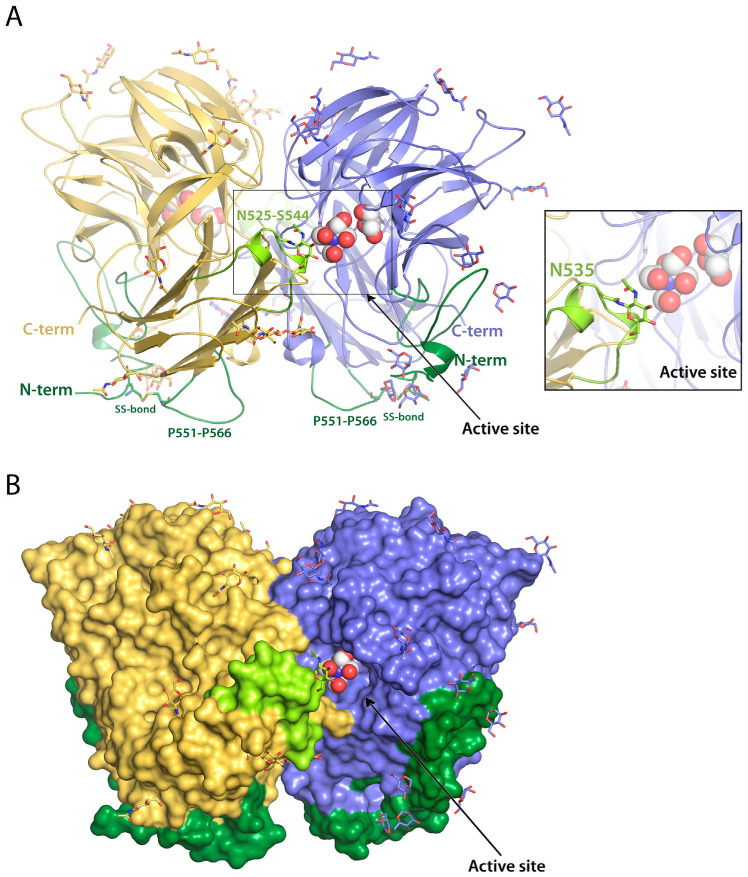
Structure of homodimeric RdINV. (**A**) Cartoon and (**B**) surface representation of wild-type RdINV, showing each subunit in different colors. The N-terminal region observed in the crystal and relevant protruding loops, including sequence insertions, are highlighted in green. Mannose and NAG units at the observed O- and N-glycosylation sites, respectively, are represented as sticks. Glycerol and Bis-Tris molecules captured bound to molecule B in the crystal are represented as spheres. The inset is a zoom of the active site.

The recombinant RdINV with the N-terminal signal peptide expressed and purified from *K. phaffii* was submitted to different deglycosylation strategies prior to the crystallization trials. A single homogeneous band with a molecular weight of 85–90 kDa was observed in the SDS gel after a combined α1-2,3,6 mannosidase/Endo H enzymatic treatment, which allowed crystal structure determination at 2.05 Å resolution. Experimental details and structural determination procedures are presented in Section 3 and in Table S1. RdINV presents a homodimer arrangement that corresponds to the two molecules described within the crystallographic asymmetric unit (Figure 1). They are associated in a “butterfly” shape to form a tight homodimer related by a well-defined two-fold symmetry axis, similar to that previously described in the SoFfase homolog [30]. The dimensions of the dimer are 90 × 60 × 60 Å^3,^ and the total surface area is 35,470 Å^2^, with a buried surface of 4010 Å^2^ (PDBePISA server [34]). The interface is mostly formed by blades IV and V from each catalytic domain and the β7β8 loop from their corresponding β-sandwich domain (Figure S1) through twelve hydrogens bonds (monomer description is included in the following paragraph). Each polypeptide chain is composed of 675 residues, but the absolute lack of electron density prevented the model building of the sequence before Ser140. This issue can be attributed to the N-terminal segment up to Asp168 being a low complexity and disordered region, as predicted by DISUPRED2 and SEG [35,36], which is associated with its high number of Ser/Thr residues (Figure S1). Moreover, despite the deglycosylation treatment, many NAG or MAN moieties were visible in the electron density map. At least six O-glycosylation sites (Thr146, Ser147, Ser151, Ser153, Thr161, Thr163) located at the beginning of the chain and 13 N-glycosylation sites (Asn197, Asn240, Asn255, Asn263, Asn341, Asn350, Asn405, Asn475, Asn505, Asn514, Asn525, Asn535, Asn546) distributed over the two domains were clearly defined at the electron density map within each monomer (Figure S1). Interestingly, the O-glycosylated N-terminal region is close to the long β4β5 loop (residues Pro551-Pro566) from each corresponding β-sandwich domain within each monomer (Figure 1). Interestingly, this loop also contains a high number of Ser/Thr with seven predicted possible O-glycosylation sites. The relevance of the peculiar spatial arrangement of these two structural elements will be commented on in the next section.

**Figure 2:**
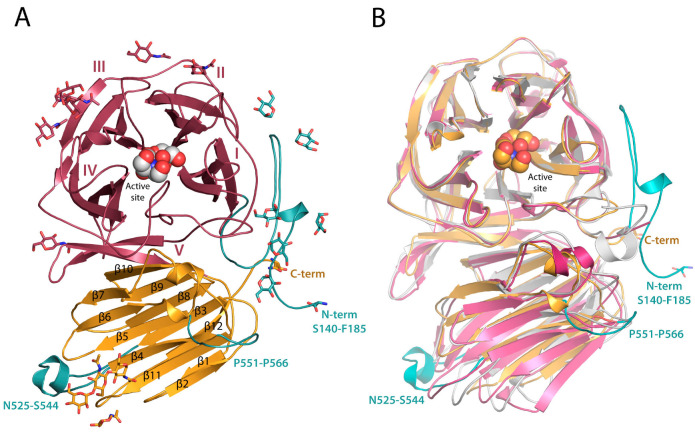
RdINV subunit folding. (**A**) The catalytic domain (blades I-V) is represented in red, and the β sandwich domain in orange. Glycerol and Bis-Tris molecules captured in the active site of molecule B are shown as spheres. Distinctive regions are shown in teal. Glycans are represented in sticks. (**B**) Structural superimposition of ScINV in pink (PDB code 4EQV) and SoFfase in grey (PDB code 3KF5) onto RdINV wild type subunit in orange, showing most different regions in teal.

RdINV subunit is composed of two domains, similar to the rest of the reported fungal β-fructofuranosidases within GH32 (Figure 2 and Figure S1). The β-propeller catalytic domain contains residues 185 to 487 and is constituted by five blades (I-V), each involving four antiparallel β-strands (ABCD) with a “W” topology (Figure 2A). The β-sandwich domain, extending from residue 495 to 673, is composed of twelve β-sheets ordered in two six-stranded clusters of antiparallel β-strands. A PEG and a glycerol molecule were captured in the catalytic site of chain A at subsites −1 and +1, whereas glycerol and Bis-Tris were trapped in chain B. A remarkable feature is the presence of a di-sulfur bridge linking Cys143 to Cys555 that connects the prominent Pro551-Pro566 loop to the visible portion of the N-terminus and possibly helps in keeping the structural integrity of the Ser140-Asp168 region that has been observed in the crystal. As it is seen in Figure 2B, the structural superimposition of RdINV to its closest homologs from yeast shows that major differences are found at the N-terminal and the Pro551-Pro566 regions commented above, but also at the loop Asn525-Ser544 that is close to the active site of the adjacent subunit within the dimer (Figure 1). These unique structural elements are made by the long inserts present in the RdINV sequence, as aligned to other fungal and bacterial enzymes (Figure S1).

### 2.2. RdINV N-Terminus Analysis

The RdINV sequence shows an unusual 168 amino acid elongation at the N-terminus when compared to the other GH32 family proteins. This region contains a 20 amino acid signal peptide and four possible N-glycosylation sites. Furthermore, and more strikingly, it is an obviously Ser/Thr-rich region (40% of its sequence) that could, therefore, contain sites of extensive O-glycosylation. About a fourth of secretory proteins were predicted to have at least one hyper-O-glycosylated region, consisting of 45 amino acids (on average) and showing more than one O-glycosylated Ser or Thr every four residues [37]. These highly O-glycosylated regions have a slight tendency to be at either end, and possible roles in conferring a specific topological configuration to the protein or in retaining it within the extracellular matrix, have been speculated [37]. It should be noted that ten putative O-glycosylation sites are predicted by the NetOGlyc server [38] in the N-terminal segment Ser140-Asp168 that is modeled in this work, and six of them have been experimentally observed in the crystal. Consequently, a similar high O-glycosylation pattern might be assumed for the remaining twenty sites predicted within the whole N-terminal elongation.

The only two other proteins that exhibit this region are the hypothetical protein C6P46_002324 from *Rhodotorula mucilaginosa* (GeneBank sequence ID KAG0663755), which is identical to RdINV, and the hypothetical protein B0A53_03777 (GeneBank sequence ID TKA53735.1) from *Rhodotorula* sp. CCFEE 5036 with 98% sequence identity. RdINV contains 675 amino acids, while other structurally resolved yeast enzymes are smaller (533 amino acids in ScINV, 512 in SoFfase and 665 in XdINV) [2,30,31]. As explained above, we observed an absolute lack of electron density for the first 139 residues in the maps corresponding to the full-sequence protein crystals. A possible explanation for this issue is the putative proteolysis of the N-terminal region during sample manipulation. Another explanation could be a high disorder of the polypeptide chain before Ser140, which is not folded over the main core of the enzyme as Ser140 is already pointing out from the protein toward the solvent (Figure 1). In any case, both situations suggest that the Ala21-Ser139 segment is not essential for the mature protein to be functional. To confirm this premise, we constructed a truncated variant of RdINV (RdINVΔ_21–139_), containing the signal peptide but without the following N-terminal 118 residues not seen in the crystal structure. RdINVΔ_21–139_ remained active, while its kinetic parameters were altered when compared to the wild type. It exhibited slightly higher *K_m_* for sucrose than RdINV (1.8-fold higher) (Table 1), while its turnover number (*k_cat_*) and catalytic efficiency increased by 3.4 and 1.8-fold accordingly. Moreover, we examined the thermal denaturation profile of RdINV and RdINVΔ_21–139_ by differential scanning fluorimetry (DSF), obtaining a similar irreversible behavior from the two samples. Therefore, we postulate that the 168 residues N-terminal elongation is not needed for proper protein folding or thermostability but, rather, may contribute to protein maturation for signaling/trafficking purposes considering its high O-glycosylation pattern. Furthermore, a look at the crystal structure of the RdINV dimer (Figure 1) suggests that the adjacent topological arrangement of the two N-terminal regions, and the corresponding Pro551-Pro566 long loops within the dimer, might configure a molecular tool to target the protein to its receptor cellular compartment or possibly to the extracellular matrix, a task assisted by the high number of O-attached glycan chains.

### 2.3. Complexes of RdINV Depict Its Active Site

As was previously reported [1], RdINV shows broad substrate specificity, hydrolyzing sucrose, 1-kestose, nystose, raffinose and inulin. However, it has a clear preference for short substrates, showing a catalytic efficiency 20–30 times superior on sucrose as compared to that on 1-kestose. To analyze its hydrolytic activity, the inactivated D188A-RdINVΔ_21–139_ mutant was deglycosylated, crystallized and used for soaking experiments with different sugars, including fructose, sucrose, 1-kestose, raffinose or nystose, and, also, ternary complexes with fructose and palatinose, trehalose or hydroxytyrosol have also been pursued. However, only complexes of this truncated variant with fructose, sucrose or raffinose were obtained, the crystals belonging to the P2_1_ space group with two complete dimers within the asymmetric unit that are essentially similar. The catalytic active site is buried in a hidden pocket 15 Å deep and 10 Å × 10 Å wide, which is accessible by a narrow slit outlined by the bulky Asn525-Ser544 loop from the adjacent subunit mentioned above (Figure 1 and Figure 2), which possibly occludes the entrance to long substrates explaining its low inulinase activity. Moreover, an N-glycosylation site has been observed at Asn535 within this protruding loop (Figure 1), and, therefore, a possible functional implication of the glycan in regulating the enzyme specificity to its natural substrate should not be discarded.

**Figure 3:**
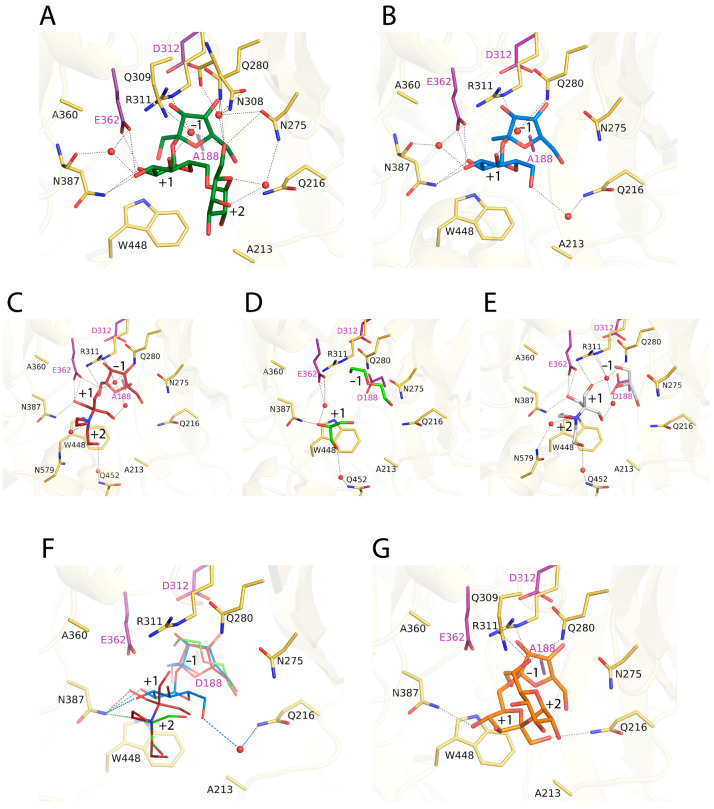
The RdINV active site. Atomic interactions of D188A-RdINVΔ_21–139_ mutant complexed with (**A**) raffinose (green), (**B**) sucrose (marine), (**C**) fructose and Bis-Tris molecules (raspberry). Detail of interactions produced at wild-type RdINV complexes with (**D**) PEG and glycerol in molecule A (lime) and (**E**) glycerol and Bis-Tris in molecule B (grey). Residues are represented in yellow sticks with the catalysts in magenta. Polar interactions are represented as dashed lines. (**F**) Structural superposition of PEG/glycerol (lime), fructose/Bis-Tris (firebrick) and sucrose (marine) complexes in the RdINV active site. Dashes lines keep the same color as the molecule involved in interaction. (**G**) Docking simulation of 6-kestose (orange) into D188A-RdINVΔ_21–139_ active site, as computed by AutoDock Vina, showing proposed polar links.

The crystal complexes show clear electron density at the active site (Figure S2) that allows unambiguous ligand modeling. Figure 3 shows the active site observed in each complex, together with the molecules trapped in the wild-type crystals commented on above.

In all complexes, the fructose at subsite −1 keeps the same atomic interactions and features previously described for other GH32 complexes [2,30], with the PEG and the glycerol molecules trapped in the wild-type crystals occupying the positions corresponding to the fructose C1, C2, C3 and C4 atoms (Figure 3C–E). Remarkably, a net of well-ordered water molecules is found in all complexes, contributing to substrate binding. On the other hand, the sucrose and the raffinose complexes are essentially identical in the common portion (Figure 3A,B), an issue that may explain that the specific hydrolytic activity observed on this three-saccharide is very similar to that reported on sucrose hydrolysis [1]. Therefore, the description of subsites +1 and +2 will be based on the complex with raffinose (Figure 3A and Figure S3). The glucose bound at subsite +1 is tightly fixed by direct hydrogen bonds to the acid/base catalytic Glu362 (through its O2), and to Asn387 (O2 and O3), with additional water-mediated links from both residues to O3. Similarly, O4 is linked to Gln280 and Arg311 through a water molecule that also makes a polar link to the O6 oxygen from galactose located at +2 subsite. This O6 participates in a well-ordered net of water molecules linking the substrate to Asn275, Gln280 and Asn308, with Asn275 being also water-linked to the galactose O4 and O5 atoms. These multiple polar links maintain a bent conformation of the raffinose three-saccharide (Figure 3A). Interestingly, the sucrose complex shows an extra interaction mediated by a water molecule connecting O6 from the glucose bound at +1 subsite to Gln216 (Figure 3B). Although this water molecule has not been observed in the raffinose complex, the interaction of the sucrose to Gln216 seems important for this substrate hydrolysis, as discussed below.

As is seen in Figure 3F, the trapped glycerol and Bis-Tris molecules are bound at the active site substituting water molecules and making many polar interactions with the protein, but all the complexes conserve the direct hydrogen bond to Asn387 observed in the sucrose and raffinose complexes at subsite +1, which supports an essential role for this residue in activity. Moreover, the Bis-Tris molecule shows one of its hydroxyl groups very close to the fructose (Figure 3C) and, thus, is in a proper position to act as an acceptor substrate in a putative transfructosylating reaction. This is consistent with and illustrates the previously reported fructose transference activity on various polyols [1].

As said before, the main product in the transfructosylating reaction catalyzed by RdINV is 6-kestose. Thus, a docking simulation with this three-saccharide was performed using AutoDock Vina [39] to depict the molecular basis of this specificity. Interestingly, as it is shown in Figure 3G, the lowest-energy solution presents a direct Asn387 link with fructose at +1 subsite through its O4, supporting the important function of this residue in the RdINV activity. Moreover, the terminal glucose moiety is fixed at subsite +2 by a net of polar interactions from its O6 to Gln280, Gln309 and Arg311 side-chains, similar to that observed in the raffinose complex. Consequently, the fructose-glucose moiety bound at subsites +1 and +2 may represent the preferred binding mode of acceptor sucrose to produce 6-kestose.

### 2.4. Design of the Mutations and Study of Their Effect on RdINV Hydrolytic Activity

A remarkable feature of the RdINV active site is the presence of Ala213 instead of a Trp that is absolutely conserved in the reported GH32 enzymes from yeast and also in many enzymes from bacteria and plants. This Trp shapes a hydrophobic platform that is defined to orient the substrate at subsites +1 and +2. Interestingly, the position corresponding to this Trp is partially occupied in RdINV by a water molecule and the adjacent Gln216 side-chain (Figure 3 and Figure S4), where a Leu or Ile is found in the homologs in fungi and bacteria providing a proper hydrophobic environment to the indole moiety. Moreover, this water molecule mediates the polar link between Gln216 and the O6 atom from sucrose (see Figure 3B). Therefore, to investigate the putative role of Ala213/Gln216, which are unique to RdINV, we constructed the A213W, Q216L, Q216T and A213W/Q216L RdINV variants. The mutants were heterologously expressed in *K. phaffii* and purified to characterize the impact of the mutations in RdINV substrate affinity, catalytic constants and hydrolytic efficiency.

All mutants followed Michaelis–Menten kinetics while using sucrose as substrate and exhibited altered kinetic parameters (Table 1) and an unfavorable change in their binding affinity (Table S2). Thus, the affinity to sucrose was affected, showing increased *Km* values when compared to the wild type, especially in the variants containing the A213W replacement, with the single and the double A213W/Q216L mutants having the most significant changes with ∼9- and 18-fold increases, respectively (Table 1). The catalytic constant was ∼2-fold higher with Q216T and A213W/Q216L and ∼2-fold lower with Q216L and A213W. However, the poorer mutants’ affinity to sucrose led to inferior catalytic efficiency in all cases, with A213W showing a significant 17-fold decrease. It seems apparent that introducing a Trp at the Ala213 position implies a detrimental steric impediment for substrate binding. Furthermore, a hydrophobic residue at the contiguous Gln216 position appears to be not well tolerated, likely by a distortion of the extended water molecules network binding sucrose to loops 207–217 and 275–280, linking the second and third β-sheets within blades I and II of the catalytic domain. In fact, the more chemically conservative Q216T replacement is the only change with a neutral effect on protein stability, according to its calculated ΔΔG value of 0.1 (Table S2).

In addition, as shown in the crystal structure of the complexes, Asn387 stands out at the RdINV active site being the sole non-catalytic residue making direct polar links to the substrates at subsite +1 (Figure 3A and Figure S3). The essential role of this position in configuring the acceptor substrate binding site was previously reported in the homolog SoFfase, where its mutation to Thr produced FOS with a broader spectrum profile than the wild type [40]. In fact, the pair Gln228/Asn254, equivalent to Ala360/Asn387 in RdINV, was considered a *hot spot* regulating the SoFfase activity, with Gln228 being mostly involved in substrate affinity, while Asn254 seems key in defining the transfer specificity. Moreover, this Gln228/Asn254 pair is structurally equivalent to the D/K signature distinguishing plant invertases from their fructosyl exo-hydrolases (FEH) counterparts, and the presence of Gln instead of an acidic residue was correlated to the SoFfase capacity to degrade long substrates. In this context, we select the Ala360/Asn387 position to investigate its role in RdINV activity and make the corresponding mutants A360Q, N387T and A360Q/N387T. As is observed in Table 1, these changes affect their activity against sucrose, increasing the *K_m_* by 3-fold, while kinetic efficiency was 30-fold decreased in both variants containing the N387T replacement, and a moderate 4-fold reduction is observed in the A360Q mutant. Consequently, the removal of Asn387 seems deleterious for sucrose hydrolysis, in agreement with its major contribution to substrate binding. However, as discussed below, it has a role in regulating transferase specificity. In addition, and contrarily to that proposed for SoFfase, the A360Q replacement did not produce a significant increase in enzyme activity against long substrates when compared to the wild type.

### 2.5. Impact of Mutations on RdINV Transfructosylating Productivity

**Figure 4:**
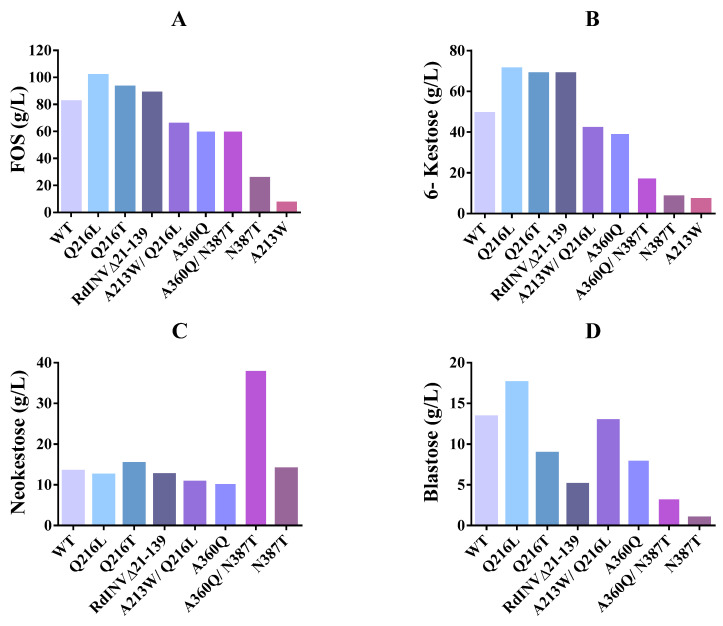
Maximum transfructosylation productivity catalyzed by the indicated RdINV variants. Evaluation of the different FOS detected in the reaction mixture of RdINV and its mutant variants at the highest FOS production point (sucrose hydrolysis ∼45–80%; time of the reaction: wt—9 h, Q216L—5 h, Q216T—6 h, RdINVΔ_21–139_—8 h, A213W/Q216L—2 h, A360Q—8 h, A360Q/N387T—3 h, N387T—8h, A213W—2 h). Reaction conditions: 600 g/L sucrose at 60 °C, using 0.5–5 U/mL of hydrolytic activity. (**A**) total FOS, (**B**) 6-kestose, (**C**) neokestose, (**D**) blastose production.

The substitutions introduced in the RdINV sequence showed some effects on the transfructosylating activity of the generated variants (Figure 4). First, the changes modify the total amount of FOS produced (Figure 4A), with increments of 13–24% in Q216/T,L and RdINVΔ_21–139_ variants, and a 20–30% similar moderate decrease in the others, except for the marked reduction observed in the N387T and A213W variants, the last dropping to a 92% reduction in FOS synthesis. This is consistent with the poor catalytic efficiency observed in sucrose hydrolysis following the removal of Asn387 or introduction of Trp at position Ala213 described above (Table 1). Moreover, the binding affinity of the acceptor sucrose must be affected by the loss of direct polar links to Asn387 at subsite +1 and also by the steric hindrance of the indole ring introduced at subsite +2 (Figure 3).

However, to investigate whether these altered activities could reflect any change in the thermostability produced by the mutations, we incubated the different variants at 60 °C for 24 h to determine irreversible thermal inactivation. This treatment showed no significant changes in their hydrolytic activity, except in the case of the variant Q216T, which reduced its activity by almost 20% after 12 h of incubation (Figure S5). However, this RdINV variant showed maximum FOS production after 6 h of reaction, as is shown in Figure S6. Therefore, a priori, we discarded the possibility that any of these enzyme variants’ activities were altered due to thermal instability at 60 °C for 24 h.

Furthermore, and in addition to the change in the total amount of FOS, different specificities are observed in the mutants. Thus, the increased FOS production described in Q216/T,L and RdINVΔ_21–139_ is basically due to a higher production of 6-kestose (Figure 4B), although the change from glutamine to threonine at position 216 changed the FOS production profile and prevented the synthesis of 1-kestose, neonystose and nystose (Figure S6). Interestingly, the Q216L and Q216T variants also differ in the production of blastose (Figure 4D). While in wild type and Q216L, an increase of blastose is observed when the production of neokestose falls, meaning that part of the blastose is formed upon its hydrolysis, in Q216T, the increase of blastose is independent of neokestose, showing that it is being synthesized by transfructosylation (Figure S6).

On the other hand, an increased neokestose amount as the major product is observed in the two variants, including the N387T substitution (Figure S6), with the double mutant A360Q/N387T producing up to 38 g/L of neokestose and exhibiting an increase of almost three-fold compared to the wild type (Figure 4C). Interestingly, the N387T change is equivalent to N254T in SoFfase, which showed a similar trend in altered FOS production profile, producing mainly neokestose instead of 6-kestose [40].

The last comment is that, as already happened with the enzyme expressed by *R. dainerensis* [32], the amount of FOS produced by all the protein variants decreased progressively after reaching its maximum values as a result of its hydrolysis. In fact, only glucose, fructose and traces of sucrose were detected in the reaction mixtures after 24 h.

**Figure 5:**
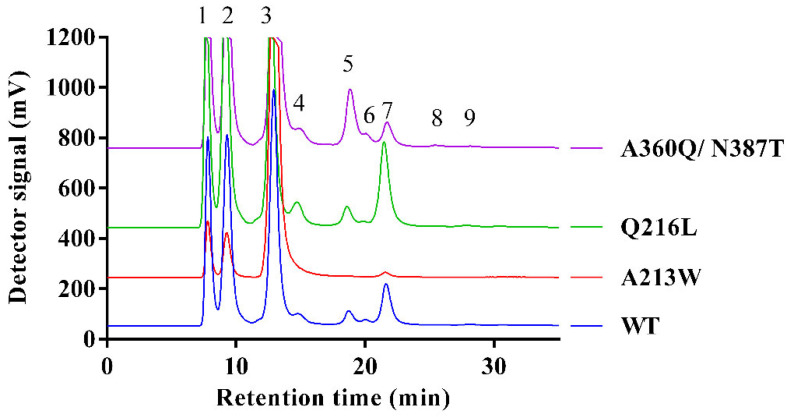
Fructosylating specificity of selected RdINV variants. Representative HPLC-ELSD chromatograms of transfructosylation reactions catalyzed by RdINV and its mutants using sucrose (600 g/L) as substrate. The reaction was carried out at 60 °C, using 0.5–5 U/mL of hydrolytic activity. Peaks: (1) fructose, (2) glucose, (3) sucrose, (4) blastose, (5) neokestose, (6) 1-kestose, (7) 6-kestose, (8) neonystose and (9) nystose.

In summary, some variants generated here exhibit redesigned specificity (Figure 5) that can be profited for synthetic purposes. Thus, the mutant Q216/L,T could be used to produce 6-kestose, while the double mutant A360Q/N387T is better to produce neokestose and the variant Q216L to obtain blastose. Moreover, even when A213W displays a poor FOS synthesis efficiency, the fact that 6-kestose appears to be the sole transference product might have an advantage in the purification step.

## 3. Material and Methods

### 3.1. Organisms, Media, Growth and Mutagenesis

β–Fructofuranosidases from *R. dairenensis* CECT 1416 (also *R. glutinis* var. *dairenensis*) and its variants were expressed in *K. phaffii* (formerly *Pichia pastoris* GS115; *his4*^−^; Invitrogen, Carlsbad, CA, USA), as referred previously [1]. It was grown in a YEP medium (1% yeast extract, 1% peptone, 2% glucose; all *w*/*v*) at 30 °C. Yeast transformants carrying the constructs based on pIB4 were selected on MD media (YNB 1.34%, biotin 4 × 10^−5^%, glucose 2%). Expression and induction of proteins in *K. phaffii* were analyzed using BMG and BMM media (both are the same as MD medium but in potassium phosphate, pH 6.0, with 1% of glycerol or 0.5% of methanol as carbon sources, respectively).

*Escherichia coli* DH5α was used for the manipulation and amplification of DNA using standard techniques. The construct (RdINV-pIB4) containing the β-fructofuranosidase RdINV gene (GenBank accession number MH779452) and the *S. cerevisiae* MFα secretion signal sequence obtained in previous work [1] was used as a template to generate all the mutants produced in this work. Site-directed mutagenesis was carried out, as mentioned before [41], using specific primers (Table S3). In the case of RdINV truncated protein, a transfer PCR was applied as referred [42] using RdINVΔ_21–139_ primers (Table S3). All the mutations were confirmed by DNA sequencing (Macrogen, Madrid, Spain).

### 3.2. RdINV Expression and Purification

*K. phaffii* transformants containing the RdINV-pIB4-derived constructs were cultivated in BMG, protein expression was induced in BMM, and heterologous activity was evaluated in culture filtrates as described previously [43]. An empty pIB4 transformant was used as a negative control. Culture filtrates containing β-fructofuranosidase activity were concentrated using 50,000 MWCO PES membranes in a VivaFow 50 system (Sartorius, Gottingen, Germany) and 50,000 MWCO PES Amicon^®^ Ultra 15 mL Centrifugal Filters (Merck. Millipore, Darmstadt, Germany). SDS-PAGE (10%) analysis confirmed protein purification, and concentration was determined using Nanodrop at 280 nm.

### 3.3. Hydrolase Activity and Kinetic Analysis

The β-fructofuranosidase hydrolytic activity was determined using the 3,5-dinitrosalycilic acid (DNS) assay adapted to 96-well microplate as described previously [1]. The reactions (50 µL) were made using 5 µL of the enzyme (diluted to fit the calibration curve) and 45 µL of 2% sucrose in 50 mM sodium phosphate buffer pH 5.5 and were incubated at 60 °C for 20 min. One unit of activity (U) was defined as that catalyzing the formation of 1 µmol of reducing sugars per minute. For kinetic analysis, the velocity was measured in triplicate using sucrose (1–200 mM). The plotting and analysis of the curves were carried out using the kinetic module of Sigma Plot (version 12). Kinetic parameters were calculated by fitting the initial rate values to the Michaelis–Menten equation. The RdINV variants’ irreversible thermal inactivation was evaluated at 60 °C since all the reactions (hydrolysis and transfructosylation) were performed at this temperature. For that, the RdINV variants were incubated in the absence of the substrate for up to 24 h. Enzyme samples were drawn after 2, 4, 6, 12 and 24 h and immediately cooled by placing them on ice. The residual activities were measured as described above (hydrolytic activity) and plotted against time.

The binding affinity change caused by mutation (ΔΔG) was calculated as logarithm of the division between catalytic efficiency of wild type and the variants.

### 3.4. Transferase Activity, Fructooligosaccharides Production and HPLC Analysis

The transferase activity of the enzymes was measured in 600 g/L sucrose and 100 mM sodium acetate pH 5.5, containing (0.5–5 U/mL) of enzymatic activity. Reactions were incubated at 60 °C in an orbital shaker (Vortemp 56, LabNet International, Woodbridge, NJ, USA) at 600 rpm as described previously (1, 32). Briefly, to evaluate the transferase productivity, aliquots of 50 µL were taken at different reaction times (0–24 h), incubated at 100 °C for 8 min, diluted in distilled water 10–20 times and filtered through 0.45 µm pore size filters (Scharlau, S.L; Sentmenat, Spain). Samples were analyzed in HPLC with a quaternary pump (Delta 600, Waters, Milford, CT, USA) coupled with a Luna NH_2_ column (4.6 × 250 mm, from Phenomenex, Torrance, CA, USA) and a precolumn-NH2 (Phenomenex, Torrance, CA, USA). The column temperature was maintained at 30 °C. Detection was performed using light scattering detector ELSD (mod. 1000, Polymer Laboratories, Ltd.; Church Stretton, UK), equilibrated at 40 °C. An automatic injector was used (mod. 717 Plus, Waters, Milford, CT, USA) with an injection volume of 10 µL. Analytes were eluted with acetonitrile/water and degassed with helium at a flow rate of 1.0 mL/min for 35 min (first 5 min acetonitrile: water 77:23, changing to 73:27 over 7 min, 73:27 for 14 min, changing to 77:23 over 1 min and staying until the end of the analysis). Data obtained were analyzed using the Waters^®^ Millennium^®^32 Software. Compounds were quantified on the base of peak areas, using the most closely related standards: fructose, glucose, sucrose and 1-kestose.

### 3.5. Crystallization and X-ray Structure Determination of RdINV

Overnight treatment (16 h) of RdINV expressed in *K. phaffii* with α1-2,3,6 mannosidase, and Endo H (NewEngland BioLabs, Hitchin, UK) at 37 °C resulted in a shift in the apparent molecular weight to 85–90 kDa. Subsequently, anionic-exchange chromatography was used to isolate the recombinant RdINV from the enzymatic deglycosylation cocktail. Several crystallization conditions were explored for purified RdINV (20 mM Tris-HCl pH 7.0, 50 mM NaCl) with high-throughput techniques with a NanoDrop robot (Innovadyne Technologies Inc., Wilmington, DE, USA) using the sitting-drop vapor-diffusion method in MRC 96-well crystallization plates (Molecular Dimensions, Sheffield, UK). Best quality crystals grew in 25% PEG 3350, 0.2 M ammonium acetate, 0.1 M Bis-Tris pH 5.5, from JCSG + Suites (Jena Bioscience, Jena, Germany), using 8 mg/mL protein concentration and 2:1 (protein: reservoir) rate. For data collection, different crystals were transferred to mother liquor solution supplemented with 15% glycerol and captured in liquid nitrogen for cryocooling. Diffraction data to 2.07 Å resolution was collected at the ALBA synchrotron station (Barcelona, Spain), and the images were processed and merged using XDS [44] and AIMLESS from the CCP4 package [45], respectively. RdINV was indexed in the C222_1_ space group, with two molecules in the asymmetric unit and 37.4% solvent content. The structure of RdINV was solved by molecular replacement using MOLREP included in CCP4 Suite [46] and ScINV [31] (39.6% sequence identity; PDB code 4EQV) as template model. Owing to lack of electron density, the 139 initial amino acids were not modeled. Crystallographic refinement was performed using REFMAC within the CCP4 suite and Phenix.refine within PHENIX [47] using local non-crystallographic symmetry (NCS) [48] and completed with the program Coot [49] combined with additional rounds of refinement.

In order to capture different RdINV complexes, the truncated D188A-RdINVΔ_21–139_ deactivated mutant lacking residues 21 to 139 was expressed, and the wild-type deglycosylation and crystallization strategy were followed. Best D188A-RdINVΔ_21–139_ crystals were grown at 5.1 mg/mL. To obtain D188A-RdINVΔ_21–139_ fructose complex, crystals were grown in 18% PEG 3350, 0.2 M MgCl_2_, 0.1 M Bis-Tris pH 5.5 solution (1:1 rate) in the presence of 20 mM fructose. To obtain D188A-RdINVΔ_21–139_ sucrose complex, a crystal grown in the same conditions was transferred for 1 h 15 min to a soaking solution containing mother liquor and 30 mM sucrose. To obtain the D188A-RdINVΔ_21–139_ raffinose complex, a crystal grown in 20% PEG 3350, 0.2 M NaCl, 0.1 M Bis-Tris pH 5.5 (2:1 rate) was transferred for 2 h to a soaking solution containing mother liquor supplemented with 30 mM raffinose. Finally, these crystal complexes were subsequently put into a fresh drop supplemented with 20% glycerol prior to cooling in the nitrogen system. Diffraction data of D188A-RdINVΔ_21–139_ in complex with fructose, sucrose and raffinose were collected at the ALBA synchrotron station (Barcelona, Spain), being indexed in the P2_1_ space group with four molecules in the asymmetric unit, at 1.86 Å, 2.38 Å and 2.27 Å, respectively. Crystallographic refinement was based on wild-type RdINV structure, and the figures were generated with PyMOL (PyMOL Molecular Graphics System, Version 2.0, Schrödlinger, LLC, USA) [50]. A full summary of data collection, data reduction statistics, and final refinement solution is provided in Table S1.

### 3.6. Automated Docking of 6-Kestose into D188A-RdINVΔ_21–139_

Autodock Vina was used for docking 6-kestose into the D188A-RdINVΔ_21–139_ active site. The ligand was manually built in Coot [49], and AutoDock Tools [51] was used to prepare the macromolecule coordinates, including polar hydrogens in the structure and fixing 9 torsions in the ligand. A grid box with 30 × 36 × 26 Å^3^ dimensions was defined by centering on the ligand within the active site. AutoDock Vina default parameters were fixed, keeping the exhaustiveness at a value of 8 [39]. Twenty solutions were calculated with binding energy ranging between −8.2 and −5.9 kcal/mol. The lowest energy conformation in the Autodock Vina result corresponds to the best solution considering chemical and binding criteria. The interactions were evaluated with PyMOL [50].

### 3.7. Differential Scanning Fluorimetry

NanoDSF experiments were performed with RdINV wild type and RdINVΔ_21–139_ variant. Intrinsic protein fluorescence at 330 and 350 nm was measured using 10 μL of sample (0.2 mg/mL of protein in 50 mM potassium phosphate buffer pH 6.0) for each capillary measurement and a ramp from 35 °C (308 K) to 95 °C (368 K) in 3 min using the Tycho NT.6 equipment (NanoTemper Technologies, München, Germany) and following the protocol optimized by the manufacturer (Tycho NT.6 Application Guide). The measurements for both samples were performed in duplicate. The fluorescence recorded during the thermal melt was plotted as 350/330-nm ratio, and the transition between folding and unfolding state, known as the inflection temperature (Ti) parameter, was automatically calculated by Tycho NT.6 software. The reversible/irreversible behavior of the wild type and the truncated variant was performed by running a thermal melt of each sample, followed by cooling and rerunning the thermal melt in the same conditions.

## 4. Conclusions

Our structure–function study extends our knowledge of the molecular mechanisms underlying the activity of β-fructofuranosidases from yeast and brings novel details to develop the biotechnological applications of RdINV and other GH32 family enzymes. Moreover, the structural analysis suggests that its unique and highly O-glycosylated N-terminal extension might configure a molecular tool to target the dimeric protein to its receptor cellular compartment or possibly to the extracellular matrix, giving clues to elucidate its physiological function in vivo. The structure of the complexes reveals the major traits of RdINV specificity, with a narrow active site cleft hindering the approach of long substrates and Asn387 being the sole non-catalytic residues making direct polar links with them. The role of key residues has been defined by mutagenesis and kinetic analysis, and the transfructosylating specificity of the generated mutants has been depicted, delivering variants with redesigned synthetic specificity. Altogether, this study provides a promising platform to generate new engineered RdINV forms to produce novel fructosyl derivatives for particular applications.

## Figures and Tables

**Table 1:** Kinetic analysis of RdINV variants.

RdINV Mutant	*K_m_* (mM)	*k_cat_* (1/s)	*k_cat_*/*K_m_* (1/s mM)
WT	5 ± 0.1	4751 ± 25	1011 ± 10
Q216L	24 ± 1	2324 ± 13	98 ± 3
Q216T	13 ± 1	11,496 ± 182	864 ± 39
A360Q	15 ± 0.2	3814 ± 16	251 ± 21
N387T	17 ± 0.8	583 ± 5	35 ± 1
A360Q/N387T	14 ± 0.6	486 ± 4	34 ± 1
A213W	47 ± 3	2716 ± 45	58 ± 3
A213W/Q216L	88 ± 9	10,523 ± 329	120 ± 4
RdINVΔ_21–139_	9 ± 1	16,384 ± 216	1781 ± 16

Measurements were made using sucrose as a substrate. Values represent an average of three replications. The *k_cat_* values were calculated assuming a protein molecular mass of 172 kDa and 100 kDa for RdINV wild type and RdINVΔ_21–139_, respectively. The ± sign refers to standard errors based on curve fitting Michaelis–Menten model using SigmaPlot.

## Data Availability

The coordinates and structure factors of RdINV and RdINVΔ_21–139_ complexed with fructose, sucrose and raffinose have been deposited in the Protein Data Bank with the accession codes 8BEQ, 8BES, 8BET and 8BEU.

## Supplementary Materials

The following supporting information can be downloaded at: https://www.mdpi.com/article/10.3390/ijms232314981/s1; Table S1: Crystallographic statistics; Table S2: Binding affinity change caused by the mutation; Table S3: Oligonucleotides employed in this study; Figure S1: Sequence comparison of RdINV with homologs; Figure S2: Ligands bound at RdINV active site in the crystals; Figure S3: Transfructosylation activity of RdINV; Figure S4: Comparison of RdINV active site with its homologues. Structural superposition of ScINV (PDB code 4EQV, marine) and SoFfase (PDB code 3KF5, gray) onto D188A-RdINVΔ_21–139_ (yellow) complexed with raffinose (green). The catalytic residues are colored in magenta; Figure S5: Irreversible thermal inactivation of the RdINV variants at 60 °C. The protein variants were incubated at 60 °C, and then their hydrolytic activity measured after 2, 4, 6, 12 and 24 h of incubation. Data were measured in triplicate and standard errors are represented; Figure S6: Transfructosylation productivity of the RdINV variants. Progress of transfructosylation reactions of the referred RdINV variants. Reaction conditions: 600 g/L sucrose at 60 °C, using 0.5–5 U/mL of hydrolytic activity.

## Abbreviations

GH: glycosyl hydrolase; FOS, fructooligosaccharides; RdINV, β-fructofuranosidase from *Rhodotorula dairenensis*; SoFfase, *Schwanniomyces occidentalis* β-fructofuranosidase; XdINV, *Xanthophyllomyces dendrorhous* β-fructofuranosidase; ScINV, *Saccharomyces cerevisiae* invertase.
