# Supplementary material for: Insights into the Structure of the Highly Glycosylated Ffase from Rhodotorula dairenensis Enhance Its Biotechnological Potential
Source: Int J Mol Sci. 2022 Nov 29;23(23):14981. doi: 10.3390/ijms232314981 (PMC9741242; doi:10.3390/ijms232314981)
Supplement: Supplementary file 1 [file ijms-23-14981-s001.zip › ijms-2023786-supplementary.pdf]

SUPPLEMENTARY MATERIAL

Insights into the structure of the highly-glycosylated Ffase from *Rhodotorula dairenensis* enhance its biotechnological potential

Elena Jiménez-Ortega[a^], Egle Narmontaite[b^], Beatriz González-Pérez[a], Francisco J. Plou[c] María Fernández-Lobato[b*] and Julia Sanz-Aparicio[a*]

[a] Department of Crystallography and Structural Biology, Institute of Physical-Chemistry Rocasolano, CSIC, 28006 Madrid, Spain
[b] Department of Molecular Biology, Centre of Molecular Biology Severo Ochoa, CSIC-UAM, 28049 Madrid, Spain
[c] Institute of Catalysis and Petrochemistry, CSIC, 28049 Madrid, Spain

*Corresponding authors: xjulia@iqfr.csic.es; mfernandez@cbm.csic.es
^These authors contributed equally to this work

Table S1. Crystallographic statistics (values in parentheses are for the high-resolution shell).

**Crystal data**	InvRd wt PEG/GOL, GOL/Bis-Tris	D188A-RdINVΔ_{21-139} /FRU/Bis-Tris	D188A-RdINVΔ_{21-139} /SUC	D188A-RdINVΔ_{21-139} /RAF
Space group	$C222_1$	$P2_1$	$P2_1$	$P2_1$
Unit cell parameters				
a, b, c (Å)	103.38, 108.24, 196.60	80.85, 112.69, 139.62	80.34, 112.81, 139.08	81.01, 114.54, 139.16
α, ß, γ (°)	90.00, 90.00, 90.00	90.00, 104.89, 90.00	90.00, 104.74, 90.00	90.00, 104.58, 90.00
**Data collection**				
Beamline	ALBA XALOC	ALBA XALOC	ALBA XALOC	ALBA XALOC
Temperature (K)	100	100	100	100
Wavelength (Å)	0.979257	0.979257	0.979264	0.979264
Resolution (Å)	47.41 -2.07 (2.12 - 2.07)	45.70-1.86 (1.89 – 1.86)	45.65 - 2.38 (2.42 - 2.38)	46.25 – 2.27 (2.31 – 2.27)
**Data processing**				
Total reflections	367663 (23880)	1122695 (57382)	647026 (33979)	775924 (39278)
Unique reflections	66921 (4459)	202338 (10039)	96053 (4777)	113410 (5618)
Multiplicity	5.5 (5.4)	5.5 (5.7)	6.7 (7.1)	6.8 (7.0)
Completeness (%)	99.5 (99.9)	99.9 (100.0)	99.8 (99.7)	99.9 (99.9)
Mean $I/\sigma(I)$	9.9 (3.5)	14.0 (2.8)	11.8 (3.5)	9.1 (2.9)
$R_{merge}^{\dagger}$ (%)	14.5 (66.6)	7.1 (61.2)	11.4 (67.8)	13.2 (68.9)
$R_{pim}^{\dagger\dagger}$ (%)	6.9 (32.1)	3.3 (28.2)	4.7 (27.8)	5.5 (27.9)
CC1/2 (%)	99.3 (74.1)	99.8 (83.3)	99.6 (87.6)	99.3 (86.6)
Molecules per ASU	2	4	4	4
**Refinement**				
R_{work} / $R_{free}^{\dagger\dagger\dagger}$ (%)	17.17/20.92	18.48/21.42	18.43/22.22	20.61/24.70
**Nº of atoms/average B** (Å^2)				
Protein	8172/24.03	16191/27.20	16146/39.12	16133/50.68
Ligands	474/44.53	958/50.37	883/64.35	920/75.58
Water Molecules	599/29.94	1980/34.66	694/36.83	561/45.48
All atoms	9245/25.46	19129/29.14	17723/40.28	17614/51.82
**Ramachandran plot** (%)				
Favoured	96	96	96	96
Outliers	0	0	0	1
**RMS deviations**				
Bonds (Å)	0.0085	0.0050	0.0085	0.0065
Angles (°)	1.4249	1.3812	1.4391	1.4445
**PDB accession codes**	8BEQ	8BES	8BET	8BEU

$^{\dagger}R_{merge} = \sum_{hkl} \sum_i | I_i(hkl) – [I(hkl)]| / \sum_{hkl} \sum_i I_i(hkl)$. where $I_i(hkl)$ is the ith measurement of reflection hkl and $[I(hkl)]$ is the weighted mean of all measurements.
$^{\dagger\dagger}R_{pim} = \sum_{hkl} [1/(N - 1)]\ 1/2 \sum_i | I_i(hkl) – [I(hkl)]| / \sum_{hkl} \sum_i I_i(hkl)$. where N is the redundancy for the hkl reflection.

Table S2. Binding affinity change caused by the mutation (ΔΔG value above 0.5 is destabilizing).

**RdINV Variant**	**ΔΔG# (kcal/mol)**
WT	0
A213W	1.9
Q216L	1.6
Q216T	0.1
A213W/Q216L	1.4
A360Q	0.9
N387T	2.2
A360Q/N387T	2.3

Table S3. Oligonucleotides employed in this study

Mutant	Orientation	Sequence
A213W	F	CGTC**TGG**GGGAACCAGCACTGGGGAC
	R	TTCCC**CCA**GACGTACTCGAGCGGGTTGTAC
Q216L	F	GGAAC**TTG**CACTGGGGACACGCCACTTC
	R	CAGTG**CAA**GTTCCCGGCGACGTACTCG
Q216T	F	GGAAC**ACT**CACTGGGGACACGCCACTTC
	R	CAGTG**AGT**GTTCCCGGCGACGTACTCG
A213W/ Q216L	F	C**TGG**GGGAAC**TTG**CACTGGGGACACGCCA
	R	G**CAA**GTTCCC**CCA**GACGTACTCGAGCGGGTTG
A360Q	F	GGACTC**CAA**TACGAATGTCCGAACTTGGTCCAGG
	R	CGTA**TTG**GAGTCCGAGCAAACCGTGGTGC
N387T	F	GATC**ACT**CCCGGCGCGCCACTCGG
	R	CCGGG**AGT**GATCGAAATGTACATGAGCCAGGCC
D188A	F	GAAC**GCT**CCGAACGGTTGCCACCG
	R	TCGG**AGC**GTTCATGAATCCCTTCGGCG
RdINVΔ_{21-139}	F	GCCATCGCCGCCGCGGCTTCAGACTCGTATGCTAGCAGCTCG TCTTGCGCTCC
	R	GATACGTGCCGTTGCGGTCG

Substitutions are marked in bold.

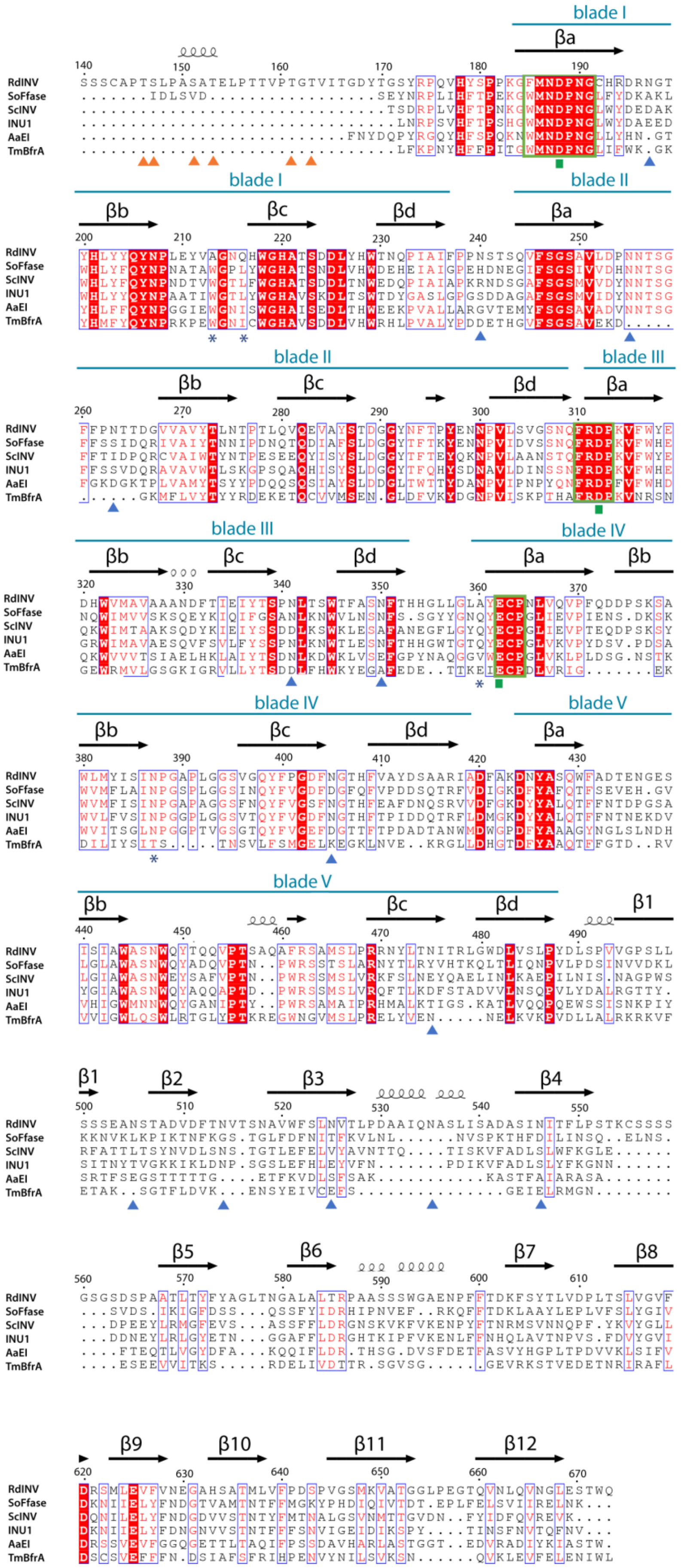
blade I
βa
RdINV
SoFfase
ScINV
INU1
AaEI
TmBfrA
blade II
βb
βc
βd
blade III
blade IV
blade V
β1
β2
β3
β4
β5
β6
β7
β8
β9
β10
β11
β12

Figure S1. **Sequence comparison of RdINV with homologues**. Structural alignment of RdINV superimposed to Ffase from *Schwanniomyces occidentalis* (SoFfase, PDB code 3KF5), Invertase from *Saccharomyces cerevisiae* (ScINV, PDB code 4EQV), Exoinulinase INU1 from *Kluyveromyces marxianus* (INU1, PDB code 6J0T), Exoinulinase from *Aspergillus awamori* (AaEI, PDB code 1Y4W) and Invertase from *Thermotoga maritima* (TmBfrA, PDB code 1UYP) using DALI server and ESPript 3.0 (http://espript.ibcp.fr) representation. Blades (I-V) from β-propeller catalytic domain are in blue. Conserved GH32 motifs are boxed in green. Green squares indicate catalytic residues and asterisks mark residues mutated in this work. O-glycosylation and N-Glycosylation sites are represented as orange and blue triangles, respectively.

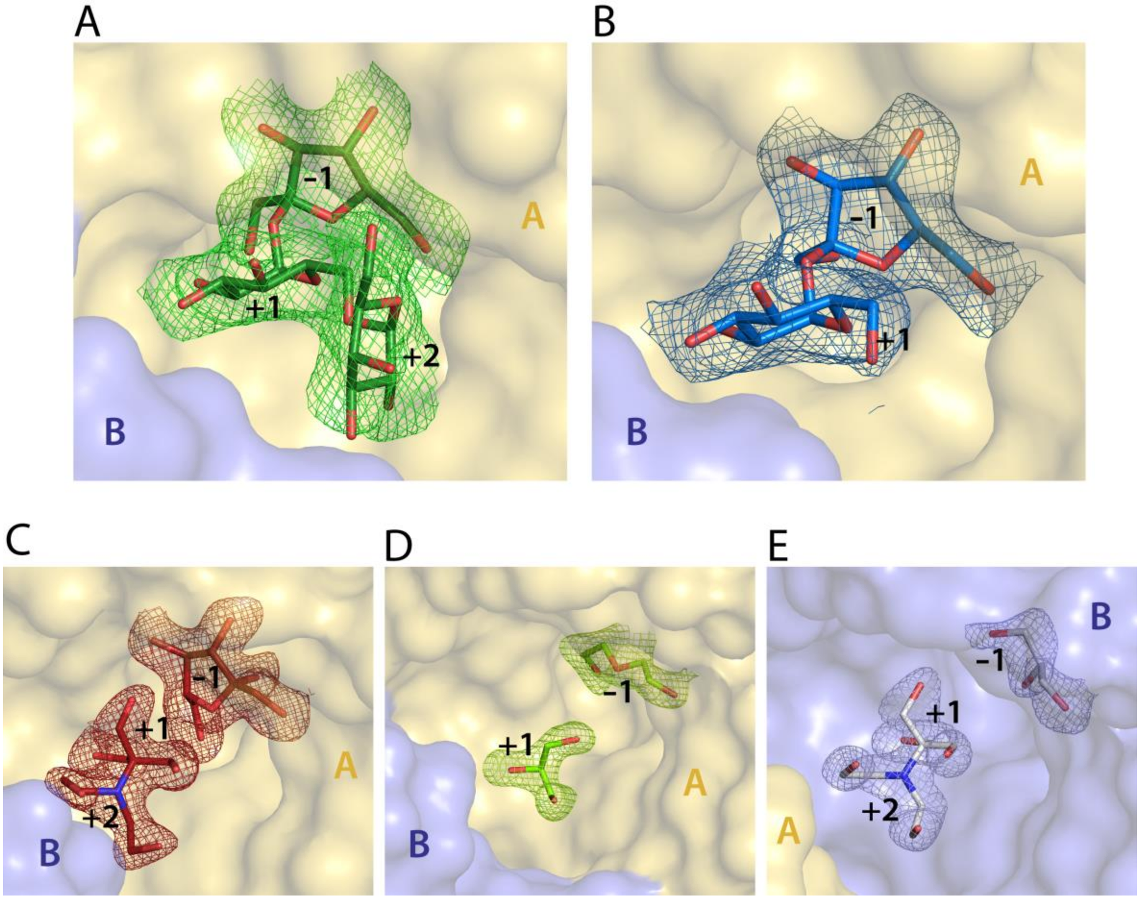

Figure S2. **Ligands bound at RdINV active site in the crystals**. Surface representation of D188A-RdINVΔ_{21-139} complexed with (A) raffinose (green), (B) sucrose (marine) and (C) fructose (firebrick). (D) Surface representation of wild type RdINV active site complexed with a PEG and a glycerol molecule (lime) and (E) a glycerol and a Bis-Tris molecule (grey). Chain A is colored in yellow and chain B in slate. The 2FoFc electron density maps are contoured at 1σ.

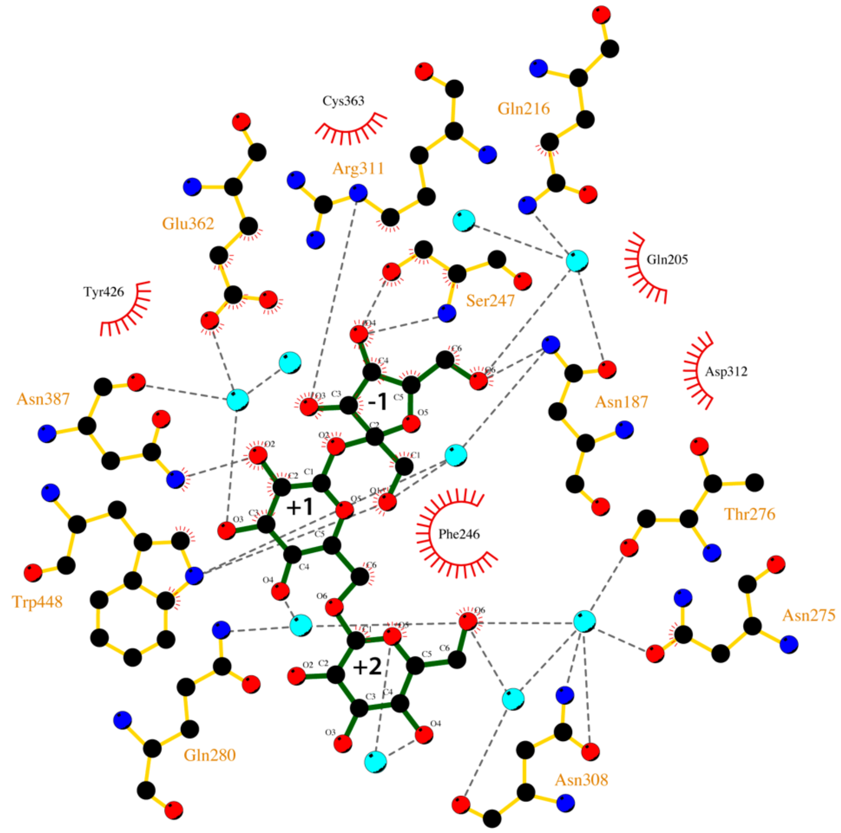

Figure S3. Schematic diagram of the atomic interactions made by raffinose at the RdINV active site. The figure was made by using LigPlot+ (Laskowski R A, Swindells M B (2011). LigPlot+: multiple ligand-protein interaction diagrams for drug discovery. *J. Chem. Inf. Model.*, **51**, 2778-2786).

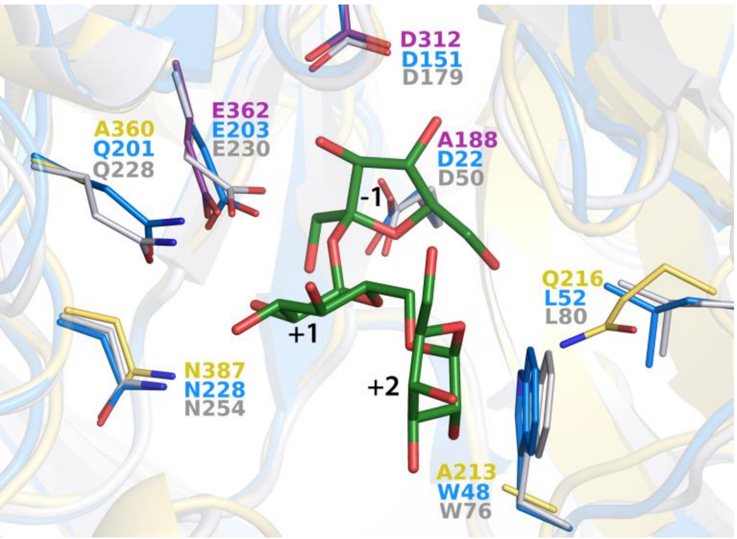

Figure S4. **Comparison of RdINV active site with its homologues**. Structural superposition of ScINV (PDB code 4EQV, marine) and SoFfase (PDB code 3KF5, gray) onto D188A-RdINVΔ_{21-139} (yellow) complexed with raffinose (green). The catalytic residues are colored in magenta.

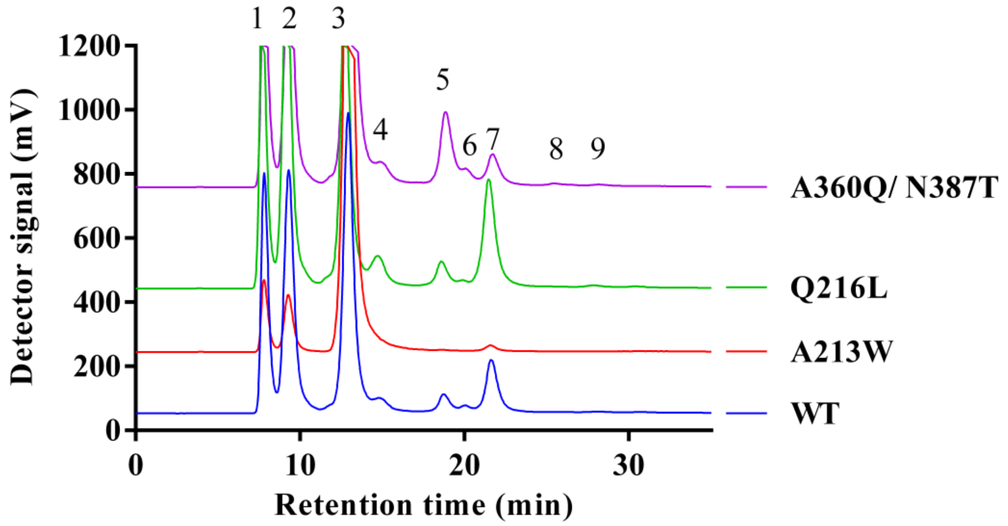

Figure S5. **Irreversible thermal inactivation of the RdINV variants at 60^{0} C**. The protein variants were incubated at 60^{0} C, and then their hydrolytic activity measured after 2, 4, 6, 12 and 24 hours of incubation. Data were measured in triplicate and standard errors are represented.

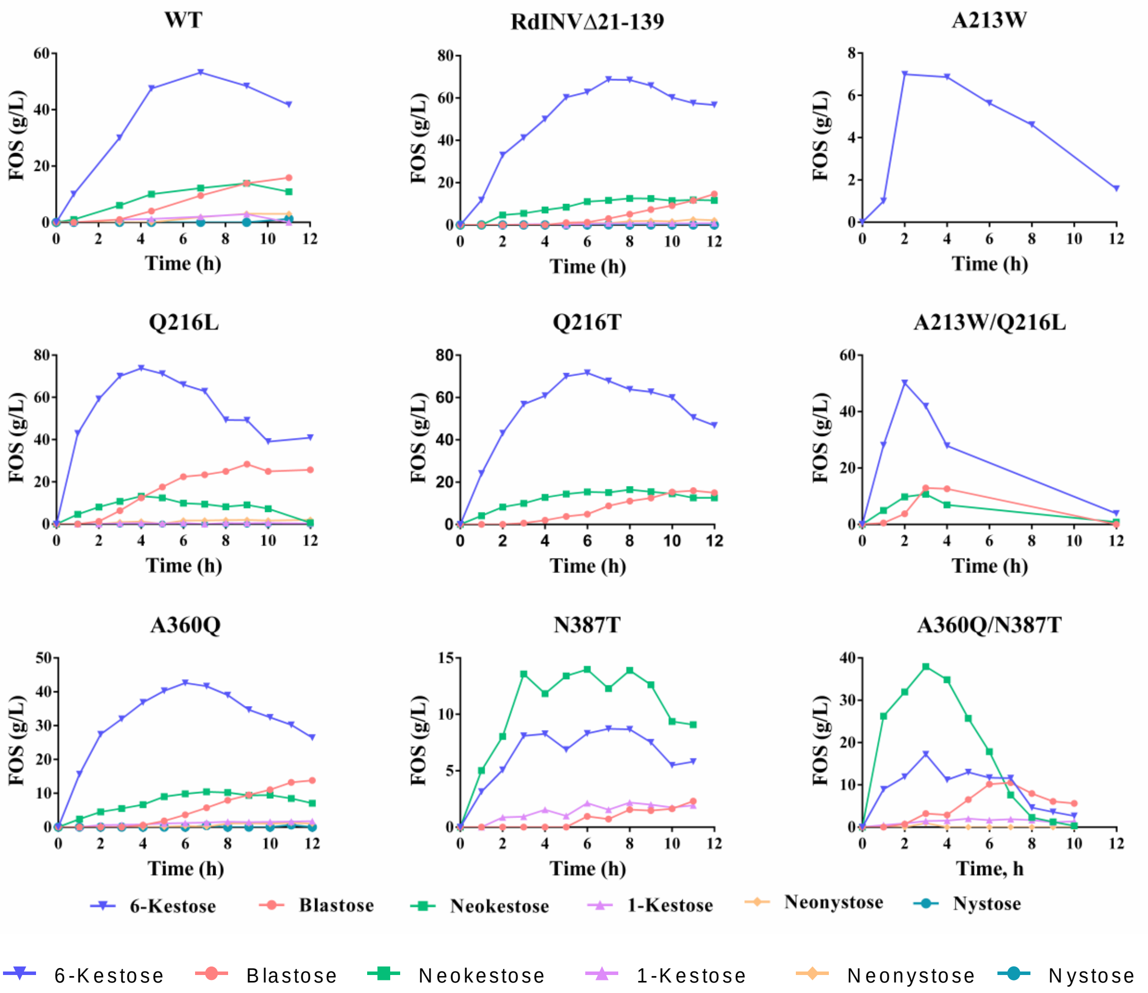

Figure S6. Transfructosylation productivity of the RdINV variants. Progress of transfructosylation reactions of the referred RdINV variants. Reaction conditions: 600 g/L sucrose at 60 °C, using 0.5- 5 U/mL of hydrolytic activity.
